# Activation of Sirtuin-1 by Pinocembrin Treatment Contributes to Reduced Early Brain Injury after Subarachnoid Hemorrhage

**DOI:** 10.1155/2022/2242833

**Published:** 2022-11-16

**Authors:** Yile Zeng, Zhongning Fang, Jinqing Lai, Zhe Wu, Weibin Lin, Hao Yao, Weipeng Hu, Junyan Chen, Xieli Guo, Xiangrong Chen

**Affiliations:** ^1^Department of Neurosurgery, The Second Affiliated Hospital of Fujian Medical University, Quanzhou, Fujian, China; ^2^Department of Neurosurgery, The Jinjiang Municipal Hospital, Quanzhou, Fujian, China; ^3^Centre of Neurological and Metabolic Research, The Second Affiliated Hospital of Fujian Medical University, Quanzhou, Fujian, China

## Abstract

Subarachnoid hemorrhage (SAH) as a devastating neurological disorder is closely related to heightened oxidative insults and neuroinflammatory injury. Pinocembrin, a bioflavonoid, exhibits different biological functions, such as immunomodulatory, anti-inflammatory, antioxidative, and cerebroprotective activities. Herein, we examined the protective effects and molecular mechanisms of pinocembrin in a murine model of SAH. Using an endovascular perforation model in rats, pinocembrin significantly mitigated SAH-induced neuronal tissue damage, including inflammatory injury and free-radical insults. Meanwhile, pinocembrin improved behavior function and reduced neuronal apoptosis. We also revealed that sirtuin-1 (SIRT1) activation was significantly enhanced by pinocembrin. In addition, pinocembrin treatment evidently enhanced peroxisome proliferator-activated receptor-*γ* coactivator expression and suppressed ac-nuclear factor-kappa B levels. In contrast, EX-527, a selective SIRT1 inhibitor, blunted the protective effects of pinocembrin against SAH by suppressing SIRT1-mediated signaling. These results suggested that the cerebroprotective actions of pinocembrin after SAH were through SIRT1-dependent pathway, suggesting the potential application of pinocembrin for the treatment of SAH.

## 1. Introduction

The outcome of aneurysmal subarachnoid hemorrhage (SAH) in clinical practice is still very poor [1–3]. SAH occurs when a cerebral aneurysm ruptures and involves a variety of pathogenic mechanisms. A great deal of preclinical and clinical researches has observed that a deteriorated inflammatory injury and heightened free-radical damage exacerbated cerebrovascular injury and might explain the poor outcome after SAH [4–6]. In addition, cerebral vasospasm and the delayed cerebral ischemia contribute to the long-term neurological deficits. Diminishing neuroinflammation and oxidative stress could also ameliorate cerebral vasospasm as well as the delayed cerebral ischemia after SAH [7, 8]. Thus, targeting cerebral inflammatory injury and free-radical insults would improve brain recovery after SAH, but effective therapies are lacking.

Increasing evidence has indicated that medicinal plants and their active ingredients may help to find new promising therapeutic drugs for central nervous system (CNS) diseases. Pinocembrin, a natural compound distributed in propolis, shows immunomodulatory, anti-inflammatory, antifree radical, and anticytotoxicity properties [9]. A great deal of research has demonstrated the promising effects of pinocembrin against various CNS diseases including ischemic brain injury, hemorrhagic brain injury, traumatic brain injury, psychiatric disorders, and neurodegenerative disorders [9–12]. In a model of intracerebral hemorrhage, pinocembrin significantly mitigated hemorrhagic brain injury by suppressing toll-like receptor 4 and reducing M1 microglia [9]. In another study, pinocembrin reduced neuronal damage in hippocampus and improved cognitive function by inhibiting autophagy in a cerebral ischemia/reperfusion model [13]. Meanwhile, numerous studies have indicated that pinocembrin could pass the blood-brain barrier [9, 14]. Due to its different pharmacological functions and no associated toxicities, pinocembrin has great potential for treating neurovascular diseases. However, much less is known about the efficacy of pinocembrin in SAH and its implicated mechanisms.

Sirtuin-1 (SIRT1) is a histone deacetylase that is widely distributed in cerebral cortex. Accumulating preclinical evidence has indicated that SIRT1 is a promising molecular candidate for SAH [15–17]. By deacetylating a variety of intracellular targets such as peroxisome proliferator-activated receptor-*γ* coactivator (Pgc-1*α*) and ac-nuclear factor-kappa B (ac-NF-*κ*B), SIRT1 provides protection in reduction of inflammatory injury, free radical damage, and cell death [18, 19]. Intriguingly, pinocembrin is able to modulate SIRT1 signaling in many diseases [20, 21]. However, it remains unknown the effects of pinocembrin on brain tissue damage caused by SAH and whether pinocembrin can activate SIRT1 and its downstream targets. Therefore, we examined whether pinocembrin protects against SAH insults and focused on SIRT1-depdent pathway.

## 2. Materials and Methods

### 2.1. Animals and In Vivo Model

All experimental studies were conformed to the ARRIVE guidelines [22]. Adult male-SD rats (weighing 250-300 g) were acquired from the Animal Center of Fujian University. Rat model of endovascular perforation SAH was built in accordance with previous protocols [16, 23]. Before SAH, 1% sodium pentobarbital was administered by intraperitoneal injection. A marked 4-0 filament was employed to puncture the origin of the left middle cerebral artery from the left internal carotid artery. Sham-treated animals received a similar procedure without vessel perforation. Following surgery, all rats were administered with buprenorphine (0.1 mg/kg) to relieve the pain. A total of 253 rats (33 rats died and 6 rats were excluded) were used. Animal groups and mortality rates can be seen in Supplementary table 1.

### 2.2. Drug Administration

Pinocembrin (Sigma-Aldrich) was resolved in 20% hydroxypropyl-b-cyclodextrin before use. Pinocembrin (10, 20, and 40 mg/kg) or vehicle was administered by oral gavage at 2 h after surgery and then once a day [24]. Resveratrol (RSV, Sigma-Aldrich), a positive SIRT1 activator, was used as a positive control drug. RSV (60 mg/kg) was resolved in 1% DMSO and administered intraperitoneally at 2 h after surgery and then once a day. The dose of RSV and injection route were based on previous studies [25]. Ex-527 (10 mg/kg) (Sigma-Aldrich) or vehicle (1% DMSO) was administered intraperitoneally for 3 days before surgery [26]. Experiment design can be seen in Supplementary Figure 1.

### 2.3. Determination of Oxidative Stress-Related Markers

The lipid peroxidation in the brain tissue was evaluated by estimation malondialdehyde (MDA). The absorbance at 532 nm was used to determine MDA content. The endogenous antioxidants including glutathione (GSH), superoxide dismutase (SOD), and catalase (CAT) were determined in line with the manufacturers' instructions (Jiancheng Bioengineering Institute).

### 2.4. ELISA Assay

Supernatants from rat brains were assayed for rat IL-1*β*, rat IL-6, and rat ICAM-1 by specific ELISA kits. The exact protocols were conducted in line with the manufacturer's instructions. The total protein levels in each sample were assessed with BCA method.

### 2.5. Immunoblotting Analysis

The same mass of supernatants were loaded onto SDS-PAGE and transferred to PVDF membranes. The membranes were blocking and then hatched with specific Abs overnight at 4°C. After that, the secondary Abs were hatched with these membranes. ECL kits were used to reveal the protein bands. Abs used can be seen in Supplementary material.

### 2.6. Immunofluorescence Staining

Brain sections were treated following the standard procedures [5, 27]. Briefly, brain sections were fixed with 4% PFA and subsequently with 5% BSA. After that, slides were stained with primary Abs and appropriate secondary Abs. The nuclei were shown by 4,6-diamidino-2-phenylindole staining. Abs used for this experiment can be seen in Supplementary table 2.

### 2.7. TUNEL Assay

TUNEL assay (Beyotime Biotechnology) was used to determine neuronal apoptosis. Frozen brain sections were treated following the standard procedures by the manufacturer. The nuclei were revealed by staining with DAPI. The apoptotic cells were captured using a fluorescence microscope.

### 2.8. Nissl Staining and H&E Staining

Nissl staining and H&E staining were used to determine post-SAH brain tissue pathological changes. In accordance with the standard protocols [28], brain sections were stained with cresol violet or hematoxylin and then cover-slipped in Permount. The surviving neurons were observed with a light microscope.

### 2.9. Behavior Function

The neurologic deficits of SAH rats were determined by using the mNSS method [29]. The rotarod test was conducted to evaluate post-SAH motor deficits [30]. Before surgery, animals needed training for 3 days. The average time to fall off was recorded. Beam walking test was conducted blindly according to a previous study by Zhang et al. [28]. Before the normal beam walking test, rats were pretrained for 3 days.

### 2.10. Brain Water Content Analysis

As previous studies reported [31], the intact brains were quickly divided into two parts including contralateral and ipsilateral hemispheres. Each part was weighed immediately (WW) and then dried in an oven. After that, each part was weighted again (DW). Brain edema ratio = (WW − DW)/WW × 100%.

### 2.11. Statistics

Data was expressed as mean ± s.d. Student's *t*-test was used when comparing two groups. For beam walking test, two-way analysis of variance with Tukey post hoc test was conducted. One-way analysis of variance with Tukey post hoc test was conducted for other data. The GraphPad Prism was employed for statistics. Probability value < 0.05 was considered significantly different.

## 3. Results

### 3.1. Pinocembrin Mitigated Brain Edema and Neurological Impairment and Enhanced SIRT1 Expression following SAH

**Figure 1:**
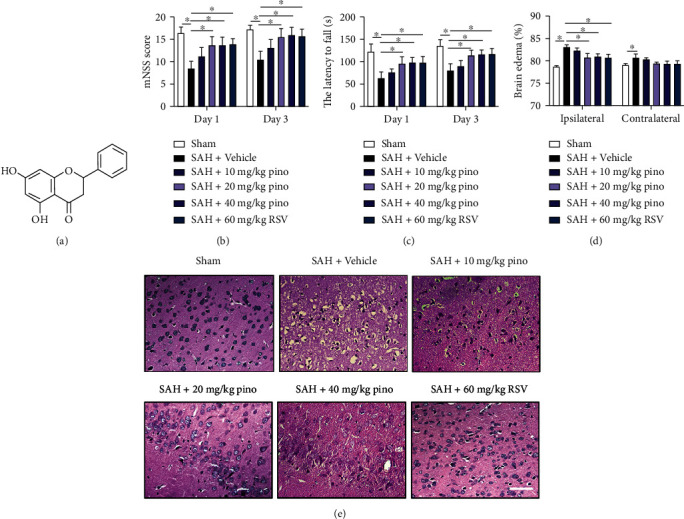
Pinocembrin improved neurological function and reduced brain edema after SAH. (a) The chemical structure of pinocembrin. The mNSS score (b) and rotarod tests (c) were analyzed at 24 and 72 h post-SAH. (d) The brain water content in all experimental groups was analyzed at 24 h post-SAH. (e) Representative photomicrographs of HE-stained images. Scale bar = 50 *μ*m. Values were presented as mean ± SD, *n* = 6-10 per group. ^∗^*P* < 0.05.

**Figure 2:**
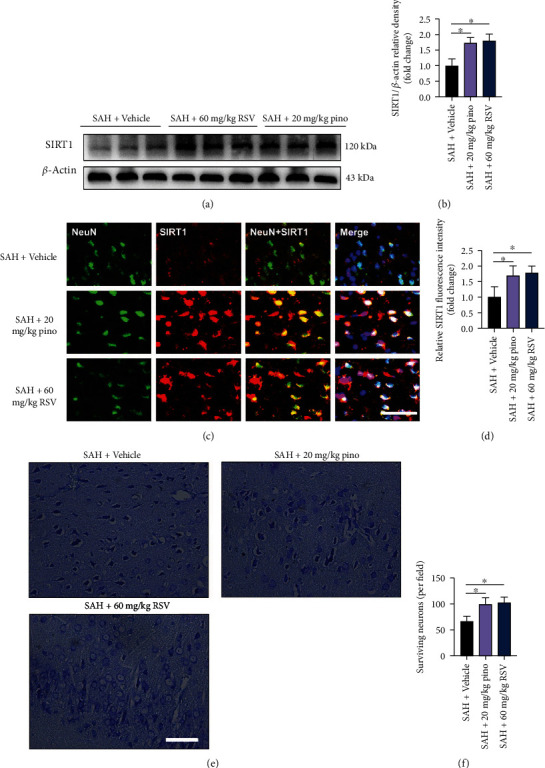
Pinocembrin upregulated SIRT1 activation and improved histological outcomes after SAH. (a) Western blotting was used to analyze SIRT1 expression. (b) Pinocembrin increased SIRT1 expression after SAH. (c) Representative photomicrographs of SIRT1 staining in brain tissue at 24 h post-SAH. (d) Pinocembrin resulted in enhanced SIRT1 staining at 24 h post-SAH. (e) Representative photomicrographs of cresyl violet-stained images. (f) Pinocembrin improved neuronal survival at 72 h post-SAH. Scale bar = 50 *μ*m. Values were presented as mean ± SD, *n* = 6 per group. ^∗^*P* < 0.05.

Accumulating evidence has indicated that RSV could significantly reduce EBI and improve neurological outcome after SAH; RSV was employed as a positive control drug.  Figure 1(a) shows the chemical structure of pinocembrin. It indicated that RSV and pinocembrin at doses of 20 and 40 mg/kg evidently decreased post-SAH neurological deficient scores and motor impairment (*P* < 0.05) (Figures 1(b) and 1(c)). But 10 mg/kg pinocembrin failed to improve post-SAH neurological outcome. Additionally, RSV and pinocembrin administration at 20 and 40 mg/kg markedly mitigated post-SAH brain edema as well as histopathological impairment (*P* < 0.05) (Figures 1(d) and 1(e)). Our data indicated that 20 mg/kg was the optimal dose after SAH. We then evaluated the effects of pinocembrin on SIRT1 expression after SAH. Western blotting data and immunofluorescence staining results showed that both RSV and pinocembrin significantly enhanced SIRT1 levels in the brain tissue following SAH (*P* < 0.05) (Figures 2(a)–2(d)). Meanwhile, Nissl staining indicated that both RSV and pinocembrin improved post-SAH neuronal survival (*P* < 0.05) (Figures 2(e) and 2(f)).

### 3.2. Pinocembrin Activated SIRT1-Mediated Signaling after SAH

**Figure 3:**
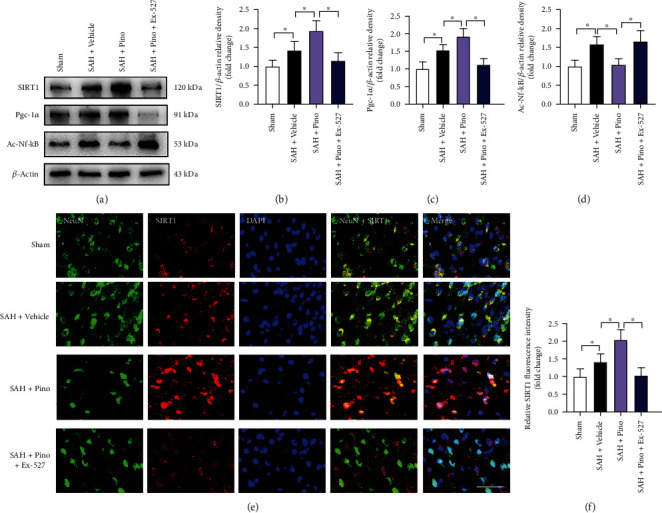
Pinocembrin activated SIRT1-mediated signaling following SAH. (a) Protein bands of SIRT1, Pgc-1a, and ac-NF-*κ*B after pinocembrin treatment. Pinocembrin upregulated SIRT1 (b) and Pgc-1a (c) levels and suppressed ac-NF-*κ*B (d) expression after SAH. (e) Representative photomicrographs of SIRT1 staining in brain tissue at 24 h post-SAH. (f) Pinocembrin resulted in enhanced SIRT1 staining at 24 h post-SAH. Scale bar = 50 *μ*m. Values were presented as mean ± SD, *n* = 6 per group. ^∗^*P* < 0.05.

Pgc-1*α*, the downstream target of SIRT1, plays a crucial in regulating oxidative metabolism and cell survival. In addition, SIRT1 can deacetylate RelA/p65 subunit of NF-*κ*B to suppress inflammatory injury. As shown, our data revealed that pinocembrin evidently enhanced the expressions of SIRT1 and Pgc1-*α* and decreased the protein levels of ac-NF-*κ*B after SAH (*P* < 0.05) (Figures 3(a)–3(d)). Ex-527 treatment validated the interaction between pinocembrin and SIRT1 signaling. It showed that Ex-527 suppressed the enhanced SIRT1 and Pgc1-*α* by pinocembrin and further aggravated post-SAH ac-NF-*κ*B expression (*P* < 0.05) (Figures 3(a)–3(d)). Similarly, immunofluorescence staining results indicated that pinocembrin enhanced post-SAH SIRT1 activation, which could be reversed by Ex-527 (*P* < 0.05) (Figures 3(e) and 3(f)).

### 3.3. Pinocembrin Inhibited Free Radical Damage after SAH

**Figure 4:**
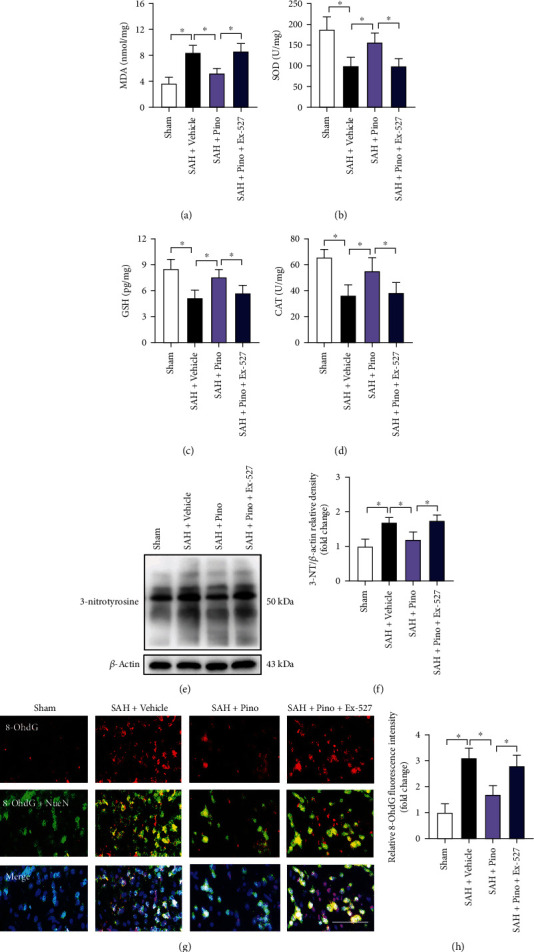
Pinocembrin alleviated free radical injury after SAH. Measurement of intracellular MDA (a), SOD (b), GSH (c), and CAT (d) after pinocembrin treatment. (e) Protein bands of 3-nitrotyrosine after pinocembrin treatment. (f) Pinocembrin suppressed 3-nitrotyrosine expression after SAH. (g) Representative images of 8-OhdG staining in brain tissue. (h) Pinocembrin resulted in decreased 8-OhdG staining at 24 h post-SAH. In contrast, Ex-527 abrogated the antifree radical effects of pinocembrin. Scale bar = 50 *μ*m. Values were presented as mean ± SD, *n* = 6 per group. ^∗^*P* < 0.05.

Free radical insults contribute greatly to SAH-induced brain injury. As shown, SAH insults aggravated lipid peroxidation and decreased the endogenous antioxidant enzyme activities. On the contrary, pinocembrin exhibited decreased levels of MDA and high intracellular endogenous antioxidant enzyme activities (*P* < 0.05) (Figures 4(a)–4(d)). Western blot and immunofluorescence staining results further showed that SAH significantly increased the formation of nitrotyrosine and 8-OhdG immunity, which could be abated by pinocembrin (*P* < 0.05) (Figures 4(e)–4(h)). In contrast, the specific inhibitor of SIRT1, Ex-527, significantly abrogated the antifree radical insults of pinocembrin after SAH (*P* < 0.05) (Figures 4(a)–4(h)).

### 3.4. Pinocembrin Inhibited Post-SAH Inflammatory Injury

**Figure 5:**
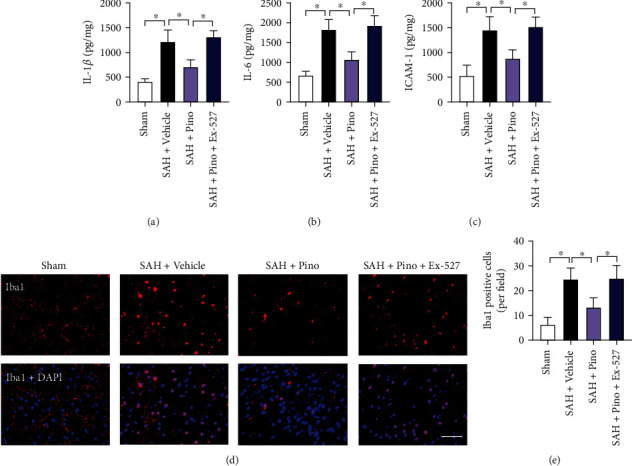
Pinocembrin mitigated post-SAH inflammatory injury. ELISA was used to detect proinflammatory cytokine release. Pinocembrin decreased the levels of IL-1*β* (a), IL-6 (b), and ICAM-1 (c) after SAH damage. (d) Representative photomicrographs of Iba1 staining in brain tissue at 24 h post-SAH. (e) Pinocembrin resulted in decreased microglia activation at 24 h post-SAH. In contrast, Ex-527 abrogated the anti-inflammatory effects of pinocembrin. Scale bar = 50 *μ*m. Values were presented as mean ± SD, *n* = 6 per group. ^∗^*P* < 0.05.

Neuroinflammation is also essential to how SAH develops. As shown, SAH markedly triggered microglia activation in the brain as well as the inflammatory cytokine secretion. In contrast, pinocembrin evidently suppressed the acute inflammatory injury after SAH (*P* < 0.05) (Figures 5(a)–5(e)). Ex-527 was administered before SAH induction. As expected, animals pretreated with Ex-527 blunted the anti-inflammatory effects of pinocembrin on SAH (*P* < 0.05) (Figures 5(a)–5(e)).

### 3.5. Pinocembrin Ameliorated Neuronal Death and Behavior Impairment after SAH

**Figure 6:**
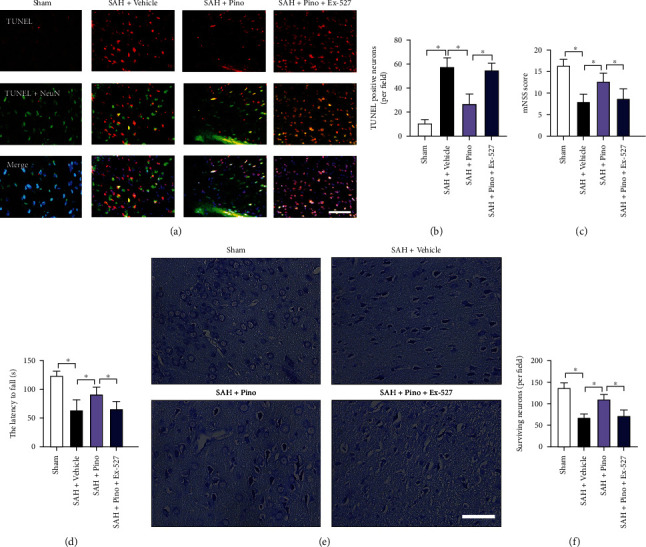
Pinocembrin reduced neuronal apoptosis and improved neurological outcomes after SAH. (a) Representative photomicrographs of TUNEL staining in brain tissue at 24 h post-SAH. (b) Pinocembrin decreased neuronal apoptosis at 24 h post-SAH. Pinocembrin improved mNSS score (c) and motor behavior (d) after SAH. Ex-527 abated the cerebroprotective effects of pinocembrin after SAH. (e) Representative photomicrographs of cresyl violet-stained images. (f) Pinocembrin improved neuronal survival at 72 h post-SAH, which could be reversed by Ex-527 pretreatment. Scale bar = 50 *μ*m. Values were presented as mean ± SD, *n* = 6-10 per group. ^∗^*P* < 0.05.

Neuronal death is closely associated with poor outcome after SAH. Both free radical insults and inflammatory injury could aggravate post-SAH neuronal apoptosis. We suspected that pinocembrin could also reduce neuronal apoptosis and improve neurological function by inhibiting oxidative and inflammatory-related damage. TUNEL staining showed that SAH dramatically aggravated neuronal apoptosis in the brain cortex, which could be statistically suppressed by pinocembrin (*P* < 0.05) (Figures 6(a) and 6(b)). Meanwhile, the aggravated neurological deficits and motor impairment by SAH could be blunted by pinocembrin (*P* < 0.05) (Figures 6(c) and 6(d)). However, all these changes were counteracted by Ex-527 pretreatment (*P* < 0.05). Nissl staining further indicated that pinocembrin improved neuronal survival subjected to SAH insults, which could be abrogated by Ex-527 (*P* < 0.05) (Figures 6(e) and 6(f)).

### 3.6. Pinocembrin Provided Long-Term Beneficial Effects after SAH

**Figure 7:**
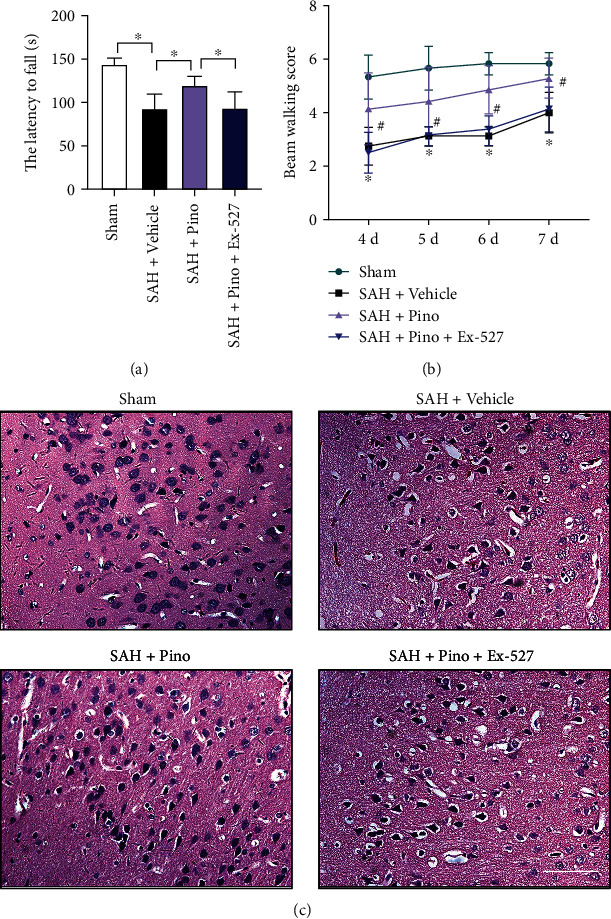
Pinocembrin improved the long-term outcome after SAH. Pinocembrin improved long-term neurobehavior function accessed by rotarod test (a) and beam walking test (b) at day 7 after SAH. In contrast, Ex-527 abated the cerebroprotective effects of pinocembrin after SAH. (c) Representative photomicrographs of H&E staining in brain tissue at day 7 post-SAH. Scale bar = 50 *μ*m. Values were presented as mean ± SD, *n* = 6-8 per group. ^∗^*P* < 0.05.

We further evaluated the long-term beneficial effects of pinocembrin after SAH. It showed that SAH insults induced significant neurological impairments at day 7 after SAH. Pinocembrin treatment significantly improved long-term neurobehavior function accessed by rotarod test and beam walking test (*P* < 0.05) (Figures 7(a) and 7(b)). In contrast, Ex-527 abated the long-term beneficial effects of pinocembrin against SAH (*P* < 0.05) (Figures 7(a) and 7(b)). H&E staining further indicated that pinocembrin reduced neuronal degeneration subjected to SAH insults, which could be abrogated by Ex-527 (*P* < 0.05) (Figure 7(c)).

## 4. Discussion

This study unraveled the efficacy of pinocembrin in a rat model of SAH. Our data showed that pinocembrin significantly mitigated behavior deterioration and brain tissue impairment after SAH as indicated by the decreased free radical insults, reduced inflammatory injury, and improved neuronal survival. Pinocembrin treatment also dramatically upregulated the concentrations of SIRT1 and Pgc-1*α* levels and suppressed ac-NF-*κ*B expression. Conversely, inhibition of SIRT1 by Ex-527 abolished the neuroprotective effects of pinocembrin and the positive effects on SIRT1-dependent pathway (Supplementary figure 2). These novel findings suggest that pinocembrin alleviated early brain damage following SAH by modulation of SIRT1-dependent pathway.

The heightened free radical insults and cerebral inflammatory response play key roles in the pathological cascade of brain damage after SAH [32–35]. The CNS is particularly vulnerability to free radical insults. One reason is that the brain tissue is rich in polyunsaturated fatty acids. Further, free radicals are verified as the elemental triggers of neurotoxicity. After SAH, the microglia are also rapidly activated to amplify the inflammatory insults. The stimulated microglia and free radicals further deteriorate neuronal damage after SAH [36]. Therefore, targeting the free radical damage and inflammatory insults might counteract early brain damage after SAH.

Pinocembrin has numerous therapeutic properties such as immunomodulatory, anti-inflammatory, antifree radical, and anticytotoxicity functions [37]. Evidence from preclinical studies has verified the cerebroprotective action of pinocembrin on different CNS diseases. For example, Lan et al. reported that pinocembrin effectively inhibited postintracerebral hemorrhage neuroinflammation via reduction of M1 microglia and inhibition of NF-*κ*B translocation [9]. Tao et al. have demonstrated that pinocembrin decreased ischemic insult-induced neuronal damage in cerebral ischemia by activation of autophagy [13]. However, little is known about the efficacy of pinocembrin in SAH model. In line with previous researches, rats treated with pinocembrin alleviated post-SAH inflammatory injury and free radical insults. Further, pinocembrin mitigated histological damages and behavior deterioration after SAH. However, the cellular and molecular mechanisms underlying pinocembrin's actions remain unknown.

Many researches have indicated that SIRT1 is a key target for treating SAH. SIRT1 plays an essential role in inflammatory and redox homeostasis [38–40]. Enhanced expression of SIRT1 could inhibit brain damage and prevent delayed cerebral ischemia in experimental SAH [41, 42]. By suppression of free radical damage, inflammatory insults, and microthrombi, SIRT1 prevents cerebral vasospasm and ameliorates neurological deficits after SAH. For example, RSV, a powerful SIRT1 activator, has been shown to prevent EBI, cerebral vasospasm, and delayed cerebral ischemia after SAH [41, 43]. In addition, a new study by Yuan et al. has demonstrated that RSV could ameliorate SAH-induced ferroptosis by activation of SIRT1 [44]. In view of these backgrounds, we used RSV as a positive control drug. Similarly, our data indicated that RSV significantly enhanced SIRT1 expression and provided protection against EBI after SAH. Intriguingly, pinocembrin is also able to modulate SIRT1-mediated signaling in other diseases. Cao et al. reported that pinocembrin ameliorated hepatocyte dysfunction-induced inflammatory response and oxidative damage through SIRT1/PPAR*α* [20]. Another observation study by Guo et al. revealed that pinocembrin protected against hepatic steatosis through SIRT1/AMPK signaling [21]. Interestingly, our experiments also revealed that pinocembrin significantly enhanced SIRT1 after SAH.

Pgc-1*α*, a downstream target of SIRT1, plays a crucial in regulating oxidative metabolism [45, 46]. Pgc-1*α* could scavenge free radical overproduction by inducing the endogenous antioxidant enzymes [47]. Additionally, Pgc-1*α* could reduce neuronal apoptosis, improve neuronal survival, and maintain the integrity of blood-brain barrier [48, 49]. However, Pgc1-*α* requires SIRT1 deacetylation to be fully activated [17].NF-*κ*B is a master regulator of neuroinflammatory injury in cerebrovascular diseases. In addition to phosphorylation, acetylation of NF-*κ*B is also involved in the inflammatory response. It reported that SIRT1 can deacetylate RelA/p65 subunit of NF-*κ*B to suppress inflammatory injury [19]. Zhao et al. also indicated that SIRT1 activation with melatonin ameliorated EBI after SAH by decreasing the acetylation of NF-*κ*B [50]. We then assessed the levels of Pgc1-*α* and ac-NF-*κ*B after pinocembrin administration. Our data indicated that pinocembrin dramatically induced Pgc1-*α* expression and inhibited ac-NF-*κ*B levels. More direct evidence validated the interaction between SIRT1 and pinocembrin. The specific inhibitor of SIRT1, Ex-527, successfully suppressed the enhanced expression of SIRT1 and Pgc1-*α* levels and decreased expression of ac-NF-*κ*B by pinocembrin. Meanwhile, the evident cerebroprotective effects of pinocembrin were also abrogated when treated with Ex-527. Together with our data, these observations indicated that by interacting with SIRT1, pinocembrin protected post-SAH inflammatory injury, free radical insults, and neuronal damage.

Our study has several limitations. Firstly, recent studies have indicated that SIRT1 could modulate microglia polarization, including inhibiting M1 microglia and promoting M2 microglia. In our study, pinocembrin inhibited microglia activation. However, the exact role of pinocembrin on different microglia phenotypes remains unknown. Secondly, a sterile neurogenic inflammation was also involved in the pathophysiology of SAH [51, 52]. It can induce mast cell activation and promote neurogenic inflammation [53]. Whether pinocembrin affect this sterile neurogenic inflammation remains unknown. Thirdly, in the present study, we mainly investigated the role of pinocembrin on SIRT1-mediated signaling pathway. In addition to SIRT1, other signaling targets might be involved in the protective effects of pinocembrin. Thus, additional preclinical experiments are still needed to clarify these issues.

## 5. Conclusion

In summary, we postulated that pinocembrin effectively mitigated early brain damage after SAH. By interacting with SIRT1, pinocembrin suppressed post-SAH inflammatory injury, free radical insults, and neuronal damage. Pinocembrin might be a promising therapeutic drug for human SAH.

## Data Availability

The data sets used in this study are available from the corresponding authors on reasonable request.

## Supplementary Materials

Supplementary Materials Supplementary Table 1: animal groups and mortality rates. Supplementary Table 2: the antibodies used in the study. Supplementary Figure 1: schematic illustration of experimental design. Supplementary Figure 2: the graphic abstract. Pinocembrin attenuated free radical insults, reduced inflammatory injury, and improved neuronal survival via the activation of SIRT1-dependent pathway after SAH.
