# Supplementary material for: Activation of Sirtuin-1 by Pinocembrin Treatment Contributes to Reduced Early Brain Injury after Subarachnoid Hemorrhage
Source: Oxid Med Cell Longev. 2022 Nov 16;2022:2242833. doi: 10.1155/2022/2242833 (PMC9683949; doi:10.1155/2022/2242833)
Supplement: Supplementary Materials — Supplementary Table 1: animal groups and mortality rates. Supplementary Table 2: the antibodies used in the study. Supplementary Figure 1: schematic illustration of experimental design. Supplementary Figure 2: the graphic abstract. Pinocembrin attenuated free radical insults, reduced inflammatory injury, and improved neuronal survival via the activation of SIRT1-dependent pathway after SAH. [file 2242833.f1.docx]

**Supplementary Table 1. Animal groups and mortality rates.**

| **Group** | **Alive** | **Died** | **Mortality Rates** |
| --- | --- | --- | --- |
| Sham | 40 | 0 | 0% |
| SAH+vehicle | 48 | 11 | 18.6% |
| SAH+10 mg/kg Pino | 16 | 3 | 15.8% |
| SAH+20 mg/kg Pino | 47 | 8 | 14.5% |
| SAH+40 mg/kg Pino | 16 | 3 | 15.8% |
| SAH+60 mg/kg RSV | 21 | 3 | 12.5% |
| SAH+20 mg/kg Pino+Ex-527 | 26 | 5 | 16.1% |

**Supplementary Table 2. The antibodies used in the study.**

| **Antibody** | **Item number** | **Company** |
| --- | --- | --- |
| Anti-SIRT1 | SC-15404 | Santa Cruz |
| Anti-Pgc-1α | Ab-176328 | Abcam |
| Anti-NF-кB | 12629 S | Cell Signaling |
| Anti-NeuN | MAB-377 | EMD Millipore |
| Anti-Iba1 | SC-98468 | Santa Cruz |
| Anti-Nitrotyrosine | 32-1900 | Invitrogen |
| Anti-8-OhdG | Ab-62623 | Abcam |


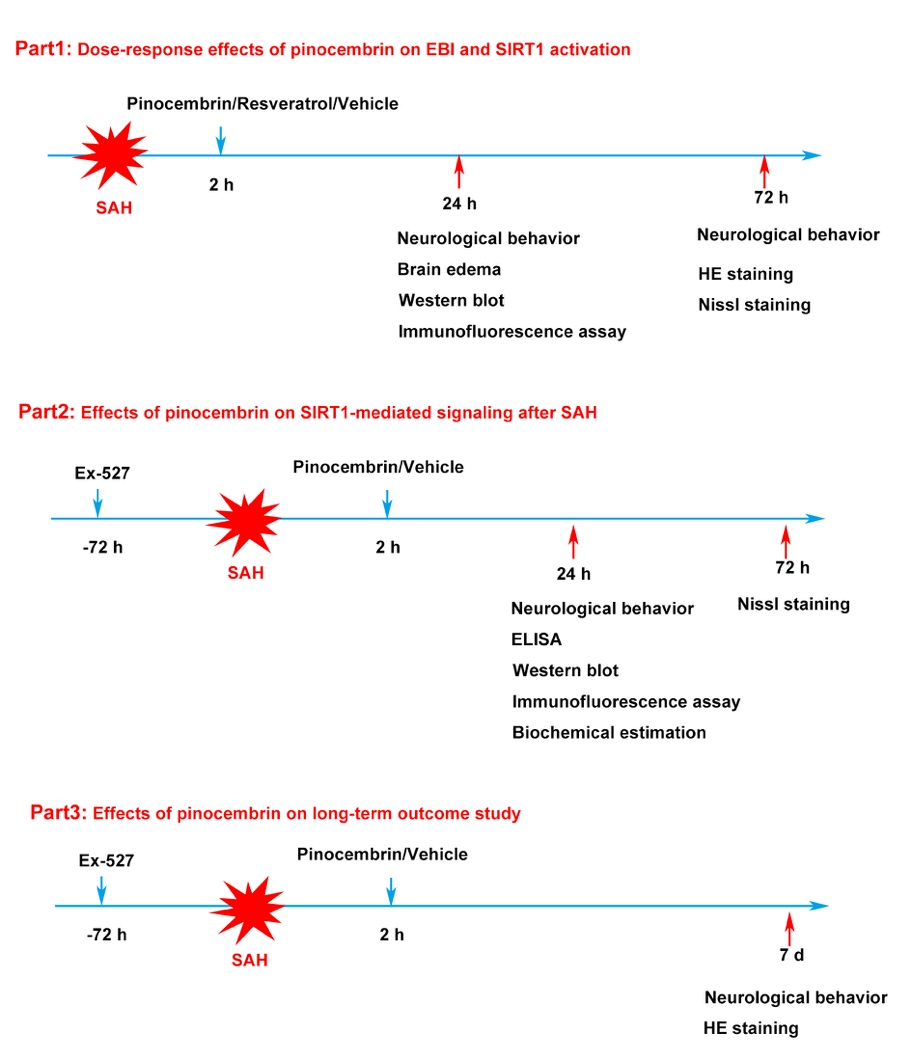


**Supplementary Figure 1.** Schematic illustration of experimental design.


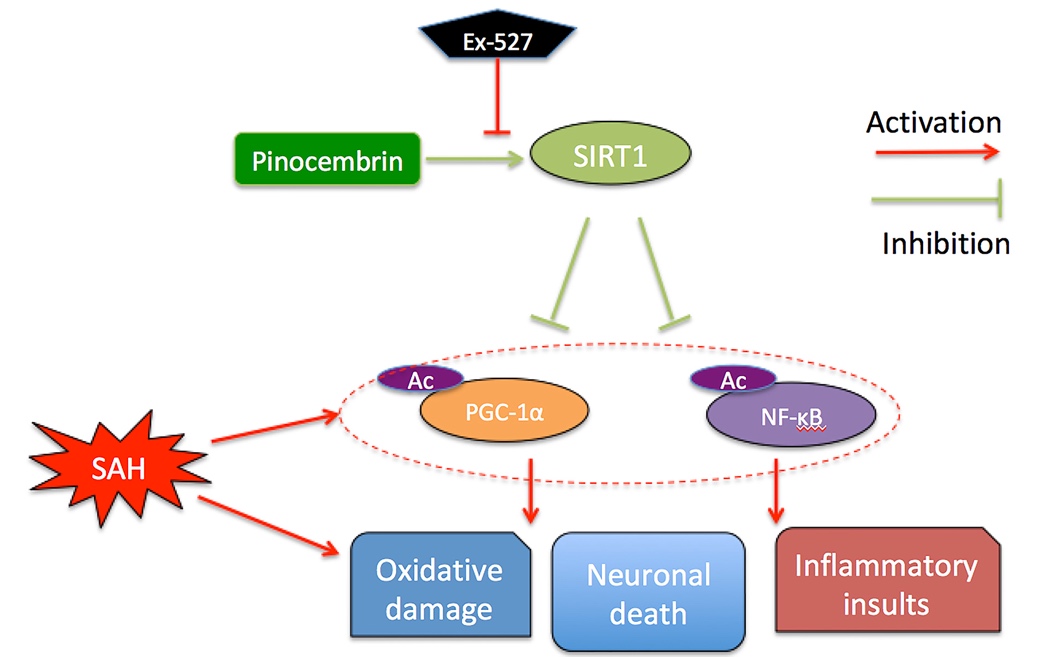


**Supplementary Figure 2.** The graphic abstract. Pinocembrin attenuated free radical insults, reduced inflammatory injury, and improved neuronal survival via the activation of SIRT1-dependent pathway after SAH.
